# Vascular health influences the correspondence between self‐report of physical activity and activity measures obtained with a smartwatch

**DOI:** 10.1002/alz.089676

**Published:** 2025-01-03

**Authors:** Riya Chaturvedi, Sophia L. Holmqvist, Marina Kaplan, Stephanie M. Simone, Molly B. Tassoni, Moira Mckniff, Tania Giovanetti

**Affiliations:** ^1^ Temple University, Philadelphia, PA USA

## Abstract

**Background:**

Greater physical activity (PA) is associated with better cognitive and vascular health, but accurate assessment of PA is challenging. Self‐report questionnaires of PA may be compared against objective measures from smartwatch sensors; the correspondence between measures may be influenced by a variety of factors such as cognition or age. The focus of this study was to identify baseline participant characteristics (vascular risk, cognition) that influence the association between self‐reported PA and PA measured with a Garmin Vivosmart 4 smartwatch in a racially diverse sample of older adults.

**Method:**

32 older adults (M age = 68.53); 32% Black/African American) with healthy cognition (n = 26) or mild cognitive impairment (n = 6) completed the International Physical Activity Questionnaire (IPAQ) to assess PA (moderate and vigorous intensity, walking; metabolic equivalent [MET] minutes) and the Framingham Stroke Risk Profile (FSRP) to assess vascular risk. A cognitive composite was evaluated as an average of t scores from several neuropsychological tests. PA was also measured over a 30‐day period in which participants wore a Garmin Vivosmart 4 smartwatch for 23 hours/day that captured duration of moderate and vigorous intensity PA and daily steps. Smartwatch measures were averaged across the 30‐day monitoring period. Discrepancy scores between the three Garmin PA metrics and three corresponding self‐reported PA measures were computed as the difference between sample‐based z‐scores. Relations between discrepancy scores and demographics/clinical characteristics were analyzed using Spearman correlations or independent samples t‐tests.

**Result:**

Greater overreporting of self‐reported walking PA was significantly related to lower education (*ρ* = ‐.440, *p* = .012) and greater vascular risk (*ρ* = .433, *p* = .013). There were no significant relations between overreporting of moderate/vigorous intensity PA and participant characteristics. There were no significant relations between cognition or sex and overreporting of PA.

**Conclusion:**

Vascular health was associated with overreporting of PA on self‐report measures relative to objective measures obtained with a smartwatch, but vascular health influenced only one of three measures (i.e., step count). Cognitive abilities and sex were not associated with discrepancies between subjective and objective measures of PA. Further work is needed to determine whether vascular heath biases either self‐report or sensor measures of daily steps in older adults.

